# Extracellular electron uptake in *Methanosarcinales* is independent of multiheme *c*-type cytochromes

**DOI:** 10.1038/s41598-019-57206-z

**Published:** 2020-01-15

**Authors:** Mon Oo Yee, Amelia-Elena Rotaru

**Affiliations:** Nordcee, Department of Biology, University of Southern Denmark, Odense, Denmark

**Keywords:** Environmental microbiology, Applied microbiology

## Abstract

The co-occurrence of *Geobacter* and *Methanosarcinales* is often used as a proxy for the manifestation of direct interspecies electron transfer (DIET) in the environment. Here we tested eleven new co-culture combinations between methanogens and electrogens. Previously, only the most electrogenic *Geobacter* paired by DIET with *Methanosarcinales* methanogens, namely *G*. *metallireducens* and *G*. *hydrogenophilus*. Here we provide additional support, and show that five additional *Methanosarcinales* paired with *G*. *metallireducens*, while a strict hydrogenotroph could not. We also show that* G*. *hydrogenophilus*, which is incapable to grow with a strict hydrogenotrophic methanogen, could pair with a strict non-hydrogenotrophic *Methanosarcinales*. Likewise, an electrogen outside the *Geobacter* cluster (*Rhodoferrax ferrireducens*) paired with *Methanosarcinales* but not with strict hydrogenotrophic methanogens. The ability to interact with electrogens appears to be conserved among *Methanosarcinales*, the only methanogens with *c*-type cytochromes, including multihemes (MHC). Nonetheless, MHC, which are often linked to extracellular electron transfer, were neither unique nor universal to *Methanosarcinales* and only two of seven *Methanosarcinales* tested had MHC. Of these two, one strain had an MHC-deletion knockout available, which we hereby show is still capable to retrieve extracellular electrons from *G*. *metallireducens* or an electrode suggesting an MHC-independent strategy for extracellular electron uptake.

## Introduction

Direct interspecies electron transfer (DIET) was discovered in an artificial co-culture of an ethanol-oxidizing *Geobacter metallireducens* with a fumarate-reducing *Geobacter sulfurreducens* where the possibility of hydrogen gas (H_2_) or formate transfer was invalidated through genetic studies^1,2^. Gene deletions rendering H_2_ and formate transfer impossible resulted in active co-cultures^1,2^, whereas deletion of genes for extracellular electron transfer proteins (EET) such as pili, and multiheme *c*-type cytochromes made the interspecies interaction impossible^1,3^. Remarkably, a deletion mutant lacking an outer membrane multiheme c-type cytochrome (OmcS) could be rescued by the addition of extracellular conductive particles, whereas a pili knock-out strain could not be rescued by conductive particles^4^. Additionally, previous studies have shown that *G*. *metallireducens* is also highly effective as an anode-respiring bacteria (ARB) generating some of the highest current densities of all *Geobacter* tested^5^. During DIET with *G*. *sulfurreducens*, *G*. *metallireducens* requires pili and certain multiheme c-type cytochromes^3^, which were also required during anode respiration^6,7^.

*G*. *metallireducens* is a strict respiratory microorganism unable to ferment its substrates to produce H_2_ for interspecies H_2_-transfer^8,9^. Consequently, *G*. *metallireducens* could not provide reducing equivalents for strict hydrogenotrophic methanogens (*Methanospirilum hungatei* and *Methanobacterium formicicum*)^10,11^. However, *G*. *metallireducens* did interact syntrophically with *Methanosarcinales* (*Methanosarcina barkeri* 800, *Methanosarcina horonobensis*, *Methanothrix harundinacea*)^10–12^ of which the last two are unable to consume H_2_^13,14^. When incubated with *Methanosarcinales*, *G*. *metallireducens* upregulated EET-proteins, and if some of these EET proteins were deleted, co-cultures became inviable demonstrating the direct electron transfer nature of the interaction^10,12^.

For co-culture incubations, *G*. *metallireducens* has been typically provided with ethanol as electron donor, but without a soluble electron acceptor. In the absence of a soluble electron acceptor, *G*. *metallireducens* releases electrons extracellularly (reaction 1) and uses the methanogen as its extracellular terminal electron acceptor (reaction 2 & 3). If electrons released by *G*. *metallireducens* cannot reach a terminal electron acceptor, than ethanol oxidation would stop, because the electron transport chain would become ineffective, and NADH produced during ethanol oxidation could not get re-oxidized.

Reaction 1. Ethanol oxidation and electron release by *G*. *metallireducens*:

$$2{{\rm{C}}{\rm{H}}}_{3}{{\rm{C}}{\rm{H}}}_{2}{\rm{O}}{\rm{H}}+2{{\rm{H}}}_{2}{\rm{O}}\to 2{{\rm{C}}{\rm{H}}}_{3}{\rm{C}}{\rm{O}}{\rm{O}}{\rm{H}}+[8{{\rm{H}}}^{+}+8{{\rm{e}}}^{-}]$$

Reaction 2. Electron uptake coupled with CO_2_ reductive methanogenesis by *Methanosarcinales*:

$${{\rm{CO}}}_{2}+[8{{\rm{H}}}^{+}+8{{\rm{e}}}^{-}]\to {{\rm{CH}}}_{4}+2{{\rm{H}}}_{2}{\rm{O}}$$

Reaction 3. Acetoclastic methanogenesis:

$$2{{\rm{C}}{\rm{H}}}_{3}{\rm{C}}{\rm{O}}{\rm{O}}{\rm{H}}\to 2{{\rm{C}}{\rm{H}}}_{4}+2{{\rm{C}}{\rm{O}}}_{2}$$

Reaction 4. Total DIET reaction by a syntrophic association between *Geobacter* and *Methanosarcinales*:

$$2{{\rm{CH}}}_{3}{{\rm{CH}}}_{2}{\rm{OH}}\to 3{{\rm{CH}}}_{4}+{{\rm{CO}}}_{2}$$

There are indications for direct interspecies electron transfer occuring in methane producing environments such as anaerobic digesters^15^, rice paddy soils^16^, and aquatic sediments^17,18^, as well as in methane consuming environments such as hydrothermal vents^19,20^. In these environments, DIET is typically inferred either because conductive materials stimulate the syntrophic metabolism but also by the co-presence of DNA and/or RNA of phylotypes related to DIET-microorganisms. DIET-pairing of *Geobacter* with methanogens was only described in two *Geobacter* species (*G*. *metallireducens*, *G*. *hydrogenophilus*)^5,10–12^. On the other hand, a series of six *Geobacter* species were unable to interact syntrophically with *Ms*. *barkeri* 800. All these *Geobacter* were modest anode respiring bacteria and did not produce high current densities at the anode^5^. Species outside of the *Geobacter*-clade have never been shown to do DIET with methanogens.

In this study, we expand the list of syntrophic-DIET pairs and investigated whether electrogenic bacteria other than *Geobacter*, could interact syntrophically with methanogens. We determined whether DIET was widespread among methanogens or was a specific trait of *Methanosarcinales* because of their high c-type cytochrome content^21^. Multiheme c-type cytochromes (MHC) were previously implicated in EET in bacteria^22^ and an MHC of *Methanosarcina acetivorans* was required for anthraquinone-2, 6-disulfonate (AQDS) respiration^23^. Here we asked whether MHC in *Methanosarcina* are required for DIET and electron uptake from electrodes.

## Materials and Methods

### Microorganisms and cultivation conditions

Cultures were purchased from the Leibniz Institute DSMZ-German Collection of Microorganisms and Cell Cultures GmbH (DSMZ) and grown on the media advised by the collection until pre-adaption to co-cultivation media.

#### Electrogens

We used the following strains of bacteria: *Rhodoferax ferrireducens* (DSM 15236), *Pelobacter carbinolicus* (DSM 2380), *Geobacter hydrogenophilus* (DSM 13691) and *Geobacter metallireducens* GS-15 (DSM 7210) available at the University of Massachusetts. For experiments with *G*. *metallireducens* strain GS15 at the University of Southern Denmark GS-15 (DSM 7210) was purchased from the DSMZ culture collection. *G*. *metallireducens*, *G*. *hydrogenophilus* and *R*. *ferrireducens* were maintained on the typical co-culture freshwater media^10^ with 55 mM ferric citrate as electron acceptor. The two *Geobacter* species were maintained with 10–20 mM ethanol as electron donor^11,12^ whereas *Rhodoferax* was maintained with 5 mM glucose. *P*. *carbinolicus* which was used as H_2_-donating strain (DIET-negative control), was pre-cultivated under fermentative conditions on 10 mM acetoin as previously described^2^.

#### Methanogens

The following strains of methanogens were tested: *Methanoculleus marisnigri* JR1 (DSM 1498), *Methanospirillum hungatei* JF1 (DSM 864), *Methanosaeta harundinacea* 8Ac (DSM 17206), *Methanothrix soehngenii* (DSMZ 2139), *Methanosarcina mazei* Gö1 (DSM 3647) and three strains of *Methanosarcina barkeri* MS (DSM 800), Fusaro (DSM 804), and 227 (DSM 1538).

*Methanothrix soehengii*, *Methanosaeta harundinacea*, *Methanospirilum hungatei* and *Methanoculleus marisnigri* were pre-grown on the typical co-culture freshwater media however without ethanol^10^ and instead containing their respective substrate 20–80 mM acetate for *Methanosaeta/Methanothrix* and H_2_:CO_2_ (80:20) for *Methanoculleus* and *Methanospirillum*.

Prior to co-cultivation, all *Methanosarcina* cultures were pre-grown on their respective substrates (20–30 mM acetate and/or 20 mM methanol) in a modified DSMZ 120c media in which tryptone was omitted and NaCl was reduced down to 1 g/L^11,12^. *M*. *mazei* (wild type and 633 k.o.) and *M*. *barkeri* 227 were pregrown with acetate and methanol, whereas *M*. *barkeri* 804 was pregrown with acetate.

A mutant strain of *Methanosarcina mazei* lacking the gene MM_0633 encoding for the multi-heme cytochrome c family protein (mutant name - *M*. *mazei* 633 k.o.) was kindly provided by Prof. Uwe Deppenmeier and Prof. Cornelia Welte. This mutant strain has been characterized in detail in Christian Krätzer’s Ph.D. thesis^24^. For the multiheme cytochrome mutant of *M*. *mazei* (633 k.o.) an antibiotic mix (ampicillin 100 µg/ml and puromycin 5 µg/ml) was added for selection and safe maintanance. This antibiotic mix was omitted in the transfer prior to co-culture inoculation and in the co-culture media.

#### Co-cultures

Co-cultures were set up without a methanogenic electron donor. As such, *Methanosarcina* co-cultures were prepared in modified DSMZ 120c media^10–12^, while co-cultures with *Methanothrix/Methanosaeta* or strict H_2_-utilizing methanogens (*Methanospirillum* and *Methanoculleus*) in modified freshwater media^10^. When the electron donating partner was *G*. *metallireducens*, *G*. *hydrogenophilus* or *P*. *carbinolicus*, 10–20 mM ethanol was provided as the sole substrate, whereas for co-cultures with *R*. *ferrireducens*, 5 mM glucose was provided as substrate.

#### Co-cultures with electrically conductive granular activated carbon (GAC)

For experiments with conductive materials, we added 25 g/L GAC (charcoal activated, Merck kGaA, Darmstadt, Germany) to the co-culture media and controls were run in parallel. No-food controls were set up to monitor for background product formation from carry-over substrates or the possible use of GAC as a food source. Single-species culture controls with and without the addition of GAC to assess the probability of ethanol oxidation using GAC as electron acceptor by a single species.

All co-cultures were incubated at 37 °C at a final volume of 10 ml and all co-cultures were set up in duplicates or more replicates.

### Analytical measurements

Samples were withdrawn anaerobically using N_2_:CO_2_ (80:20) flushed hypodermic needles to verify the levels of methane, hydrogen, acetate, ethanol, and glucose. Determination of methane (CH_4_), hydrogen gas (H_2_), ethanol and volatile fatty acids (e.g. acetate, formate) was carried out as described before^11,12^. Briefly, for experiments done at the University of Southern Denmark, gases were measured with a Trace 1300 gas chromatograph (GC) (Thermo-Scientific) equiped with a TracePLOT^™^ TG-BOND Msieve 5A column and a thermal conductivity detector (TCD). The carrier gas was argon at a flow rate of 25 mL/min with the temperatures set for the injector, oven and detector at 150 °C, 70 °C and 200 °C, respectively. To monitor ethanol, the same GC system was used with a different column, TRACE^™^ TR-Wax and a flame ionization detector (FID). The filter-sterilised liquid sample (0.5 mL) was first heated to 60 °C for 5 mins in an air-tight exetainer after which the vaporised sample was collected for measurement. The carrier gas was nitrogen flowing at 1 mL/min, with the temperatures of injector, oven and detector set at 220 °C, 40 °C and 230 °C respectively. Acetate was analysed with a Dionex^™^ ICS-1500 Ion Chromatography system, using a Dionex^™^ IonPac^™^ AS15 IC Column. The eluent was a mixture of 1.4 mM NaHCO_3_ and 4.5 mM Na_2_CO_3_ detected on an electron capture detector (ECD) at 30 mA. For experiments done at the University of Massachusetts, gases were measured by a Shimadzu 8 A gas chromatograph with a 80/100 Hayasep Q column connected to an FID. The temperatures of injector, oven and detector were set at 200 °C, 120 °C and 200 °C respectively. Ethanol was measured on a Perkin Elmer GC equiped with an Elite 5 column with helium as the carrier gas following a gradient separation protocol as follows: 50 °C for 1 min, a increase of 12 °C per minute to reach 200 °C, and a final 1.5 min at 200 °C. The temperatures for injector and detector were set at 200 °C and 300 °C, respectively.

Acetate was measured on a high-pressure liquid chromatography (HPLC) via an Aminex NPX-87H column using 8 mM H_2_SO_4_ as the eluent with a UV detector set at 210 nm. Glucose was determined at the end of the incubation, as previously described^25^ by separating on an HPLC with an Aminex Ion-Exclusion organic acid analysis column HPX-87H (Bio-Rad) and detected with a SP8430 refractive-index detector (Spectra-Physics).

### Electrochemical reactor setup

Incubations using a cathode as sole electron donor, were carried out as previously described^11^, with slight modifications. Briefly, a two-chambered H cell reactor (Adams and Chittenden, USA) with a total volume of 650 ml in each chamber was separated via a Nafion™ N117 proton exchange membrane (Ion power). The working and counter electrodes were made of graphite rods with dimensions of 2.5 × 7.5 × 1.2 cm and were connected to titanium rods. A leak-free Ag/AgCl reference electrode (3.4 M KCl) (CMA Microdialysis, Sweden). To ensure low carry over of substrates, cells were harvested in an anaerobic chamber at 4000 rpm for 10 mins, resuspended in fresh media (modified 120c – see above) prior to inoculation of the cell suspension at a final concentration of 20% in the reactor. In this set-up, the working and counter electrodes were connected via a resistor (250 Ω) and a potentiostat, however the resistor was unecessary since the cathodic potential was fully controlled by the potentiostat. The potential of working electrode was controlled with the MultiEmstat potentiostat (Palmsens, The Netherlands) set at −400 mV (vs. the standard hydrogen electrode).

## Results and Discussion

### Electrogens establish DIET syntrophy with *Methanosarcinales* methanogens

To examine the ability for direct interspecies electron uptake in new strains of methanogens, *G*. *metallireducens* was used as the default DIET-partner for co-culture experiments due to its high electrogenic capability^5^, correlated to a syntrophic ability^10,12^, which was independent of H_2_-transfer, as this *Geobacter* was incapable to produce H_2_^9,26^. We selected seven methanogenic strains as representatives of two groups: strict hydrogenotrophic methanogens (H_2_-consuming) and *Methanosarcinales-*methanogens, including H_2_-utilizing and strict non-hydrogenotrophic strains.

#### G. metallireducens paired syntrophically with five new Methanosarcinales, independent of the methanogen’s ability to consume H_2_

The electrogen *Geobacter metallireducens* was previously shown to establish DIET interactions with three species of the order *Methanosarcinales* of which two were non-hydrogenotrophic methanogens (*Methanosarcina horonobensis* and *Methanosaeta harundinacea*) and one was a H_2_-utilizing methanogen (*Methanosarcina barkeri* strain MS)^10–12^. Nevertheless, we recently reported that of two *Methanosarcina* species which are capable of DIET with *G*. *metallireducens* only one could use a cathode at −400 mV (vs. SHE)^11^ as sole electron donor. This variability in the *Methanosarcinales* capability to carry out direct electron uptake prompted us to verify whether DIET is conserved in other *Methanosarcinales*.

In this study, we evaluated five aditional strains of *Methanosarcina*, two of *M*. *barkeri* (*M*. *barkeri* 227 and *M*. *barkeri* strain Fusaro/804) two of *M*. *mazei* (Go1 and 633 k.o.) and one of the strict non-hydrogenotrophic methanogen - *Methanothrix soehngenii*.

**Figure 1:**
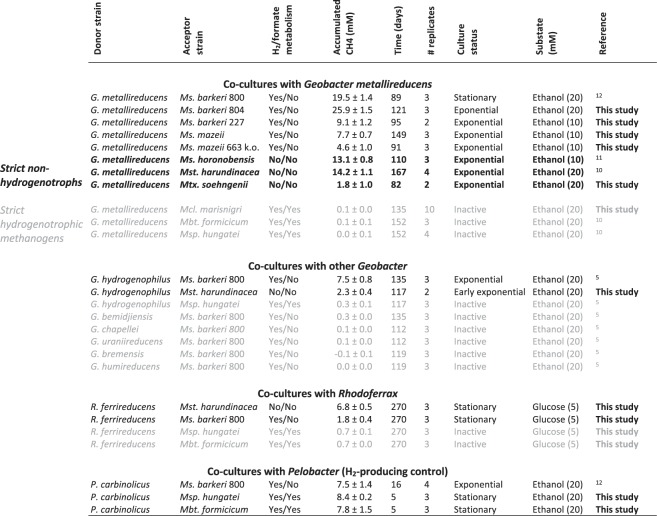
Overview of co-culture tests with 14 species of methanogens and various electroactive bacteria. As electron donors we used 5 mM glucose for tests with *Rhodoferax*; 20 mM ethanol for the rest of the co-cultures; except for tests with *M. mazei* strains, *M. horonobensis* and *M. barkeri* 227, which were provided with 10 mM ethanol.

**Figure 2:**
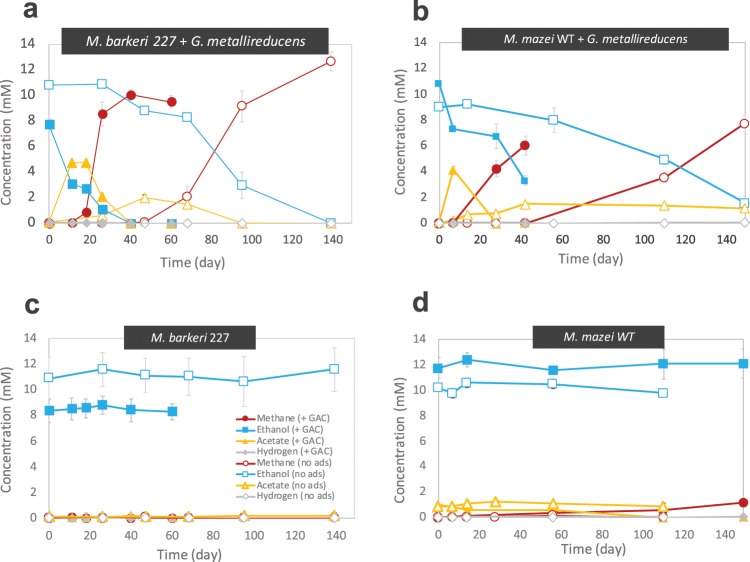
*G*. *metallireducens* co-cultivated on 10 mM ethanol together with (**a**) *M*. *barkeri* 227 and (**b**) *M*. *mazei* with or without electrically conductive particles of granular activated carbon (GAC). Control incubations of *M*. *barkeri* 227 (**c**) and *M*. *mazei* (**d**) on 10 mM ethanol as sole electron donor, with or without GAC. Changes in ethanol, acetate and methane concentrations are shown for triplicate incubations (n = 3). Whiskers represent standard deviations of the replicate incubations, and if invisible they are smaller than the symbol.

All co-cultures were provided with ethanol as electron donor in the absence of any electron acceptor other than carbon dioxide. If successful, co-cultures were anticipated to reach mid exponential growth after circa two months, in agreement to preliminary tests and previous reports on DIET-consortia^10–12^. After 75 days, all co-cultures of *G*. *metallireducens* with the five new *Methanosarcinales* effectively converted their substrate (ethanol) to products (methane) (Fig. 1). The respiratory metabolism of *G*. *metallireducens* (see reaction 1) resulted in extracellular transfer of electrons and transient formation of acetate (Fig. 2). The products of ethanol oxidation were then converted into methane (reaction 2 & 3; Fig. 2). Alone, none of the five *Methanosarcinales* converted ethanol to methane (Fig. 2).

Previously, it was shown that interactions between *G*. *metallireducens* and *M*. *barkeri* strain MS (DSM 800) could be initiated much faster and continue at a faster pace, when amended with electrically conductive materials such as granular activated carbon (GAC)^12,27^ whereas non-conductive materials such as cotton cloth had no effect^28^. GAC was shown to promote the respiratory metabolism of *G*. *metallireducens*, which oxidizes ethanol and releases electrons extracellularly onto this conductor until it reaches charge-saturation^29,30^. Afterwards, the presence of a methanogen reclaiming electrons keeps the process from coming to a halt^11^.

In this study, we subjected three additional co-cultures of *G*. *metallireducens* and *Methanosarcina* (two strains of *M*. *mazei* and one *M*. *barkeri* 227) to 25 g/L GAC to verify whether GAC can accelerate their growth. All co-cultures amended with this electrically conductive material exhibited shorter lag-phases, reached mid-exponential growth much quicker, and at a minimum tripled their methanogenesis rates (Fig. 2). On the other hand, the addition of electrically conductive particles to the three *Methanosarcina*-strains in pure culture had no impact on methane production or ethanol utilization (Fig. 2).

We have now expanded the repertoire of strains of methanogens capable of DIET with *G*. *metallireducens* by an additional five strains adding to the original three strains described in the past. Of these eight strains, three were not hydrogenotrophic. We could conclude that the capacity to carry out DIET with *G*. *metallireducens* was conserved among all tested *Methanosarcinales*, independent of their ability to use H_2_ or not.

#### *G. metallireducens* was unsuccessful to pair syntrophically with a strict hydrogenotroph - *Methanoculleus marisnigri*

Although *G*. *metallireducens* does not have the genetic possibility to form H_2_^26^, and could not evolve H_2_ when grown in pure culture on its own substrate^9^, we nevertheless previously tested whether H_2_-utilizing methanogens may have discovered a strategy to interact with this electrogenic *Geobacter*. In these former investigations we have shown that *G*. *metallireducens* was unsuccessful in developing ethanol-utilizing consortia in co-culture with two strict hydrogen-utilizing methanogens *Methanospirillum hungatei* (order *Methanomicrobiales*) and *Methanobacterium formicicum* (order *Methanobacteriales*)^10^, even in the presence of electrically conductive materials^11^.

In this study, we evaluated DIET between *G*. *metallireducens* with a third strict hydrogenotrophic species: *Methanoculleus marisnigri* (order *Methanomicrobiales*). Order *Methanomicrobiales* comprises the families *Methanomicrobiaceae* and *Methanospirillaceae*. Members of the family *Methanomicrobiaceae* (includins *Methanoculleus marisnigri*), were high in transcript abundance (13%) in rice paddies co-dominated by transcripts of *Geobacter*^16^, whereas *Methanospirillaceae* were not. This was hinting at the possibility of an interaction between *Geobacter* and *Methanomicrobiaceae*. We incubated *G*. *metallireducens* with *Mcl*. *marisnigri* for ca. 5 months, but did not observe methane production from ethanol during this timeframe (Fig. 1).

DIET evaluation by co-cultivation with *G*. *metallireducens* has now been carried out for three strict hydrogenotrophs from two major orders: *Methanomicrobiales* (*M*. *hungatei*, *M*. *marisnigri*) and *Methanobacteriales* (*M*. *formicicum*), showcasing the inability of strict hydrogenotrophic methanogens to pair with *G*. *metallireducens* (Fig. 1).

#### *G. hydrogenophilus* paired syntrophically with a second *Methanosarcinales*

Previously, we demonstrated that a second *Geobacter* species (*G*. *hydrogenophilus*) paired syntrophically with *M*. *barkeri* strain MS (DSM800). This was the only other *Geobacter* out of seven strains tested which exhibited the highest current density on the anode, similar to *G*. *metallireducens*^5^. Unlike *G*. *metallireducens*, *G*. *hydrogenophilus* does produce H_2_ during respiration^9^, but could not pair with the strict H_2_-utilizing methanogen – *Methanospirillum hungatei*^5^.

**Figure 3:**
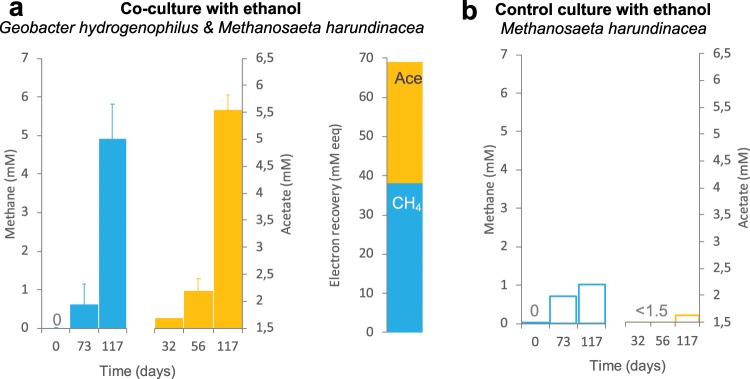
*G*. *hydrogenophilus* in co-culture with the non-hydrogenotrophic methanogen *Methanosaeta harundinacea* (**a**) produced acetate and methane when provided with 20 mM ethanol (n = 2). Electron recoveries from day 32 to day 177, showed circa half recovery of the electrons from ethanol as acetate and the other half as methane. 1 mM methane and 1 mM acetate contain 8 mM electron equivalents (eeq) according to the stoichiometry of the following reactions: CH_4_ + 2H_2_O → CO_2_ + [8e^−^ + 8H^+^]; CH_3_COOH + 2H_2_O → 2CO_2_ + [8e^−^ + 8H^+^]. (**b**) Methane and acetate in a parallel control experiment with *Methanosaeta harundinacea* provided with 20 mM ethanol (n = 1). Carry over acetate of ca. 1.5 mM is typical for *Methanosaeta*. Here we verified whether there was an effective increase in acetate due to ethanol fermentation, above the typical methane production from acetate carry over. Whiskers represent standard deviations of the replicate incubations, and if invisible they are smaller than the symbol.

*G*. *hydrogenophilus* was only tested with strains that did have the possibility to use H_2_ and it was never tested whether it can pair syntrophically with strict non-hydrogenotrophic methanogens. In this study, we tested whether *G*. *hydrogenophilus* can pair syntrophically with the non-hydrogenotrophic methanogen *Methanosaeta harundinacea* (order *Methanosarcinales*). Methane production and acetate accumulation from ethanol were used as a proxy for the efficiency of the interaction. Previously, we have shown that acetate and methane can only accumulate in successfully paired co-cultures. For example, control tests with *Methanosaeta* could not sustain methane production and acetate accumulation from ethanol in the absence of a functional ethanol-oxidizing *Geobacter*^10^. In this study, we show that over the course of 117 days, co-cultures of *G*. *hydrogenophilus* and *Methanosaeta harundinacea* accumulated 5-times more methane and 4-times more acetate than a control culture of *Methanosaeta* on ethanol run in paralel (Fig. 3). However, a small amount of methane (ca. 1 mM) was produced by *Methanosaeta* in the control culture likely due to cells being transferred together with traces of acetate (an effective substrate for this methanogen). This was consistent with previous observations^10^.

Moreover, the co-culture of *G*. *hydrogenophilus* and *Methanosaeta harundinacea* produced 9-times more methane than co-cultures with *Methanospirillum* run in parallel (Fig. 1, p = 0.03). *Methanospirrilum* was previously shown not to pair successfully with *Geobacter hydrogenophilus*^5^. Here we have shown additionally that that *G*. *hydrogenophilus* favors pairing with a strict non-hydrogenotrophic methanogen (*Methanosaeta harundinacea*) over a strict hydrogenotroph (*Methanospirillum hungatei*). This supports the notion that while *G*. *hydrogenophilus* can produce H_2_^9^ it is an ineffective H_2_ donor, unlike the H_2_-donating syntroph - *P*. *carbinolicus*^12^ (Fig. 1).

#### *Rhodoferrax ferrireducens* paired syntrophically with *Methanosarcinales* but not with strict hydrogenotrophic methanogens

**Figure 4:**
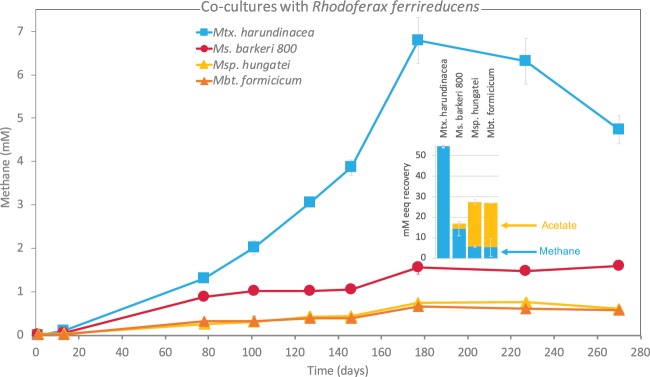
Co-cultures of four species of methanogens with *Rhodoferax ferrireducens*. Methane profiles in co-cultures provided with 5 mM glucose as sole electron donor. (inset) Estimated electron recoveries into products (acetate and methane). Incubations were carried out in triplicate (n = 3). Whiskers represent standard deviations of the replicate incubations, and if invisible, they are smaller than the symbol.

With only two out of seven *Geobacter* species showing aptitude for DIET with *Methanosarcinales*, we verified whether other highly effective electrogens outside the *Geobacter* clade could pair syntrophically via DIET. We tested this possibility for an effective anode respiring *Betaproteobacteria* - *Rhodoferax ferrireducens*^25^. Interestingly, *Rhodoferax* was predicted to outcompete *Geobacter* in a subsurface environment with low substrate flux and relatively high ammonia^31^, where non-hydrogenotrophic *Methanosarcina* species co-exists^32^. The co-existence of *Rhodoferax*, *Geobacter* and *Methanosarcina* was also noted in coastal Baltic Sea sediments^32^. It is possible *Methanosarcina* species could receive DIET-electrons from *Rhodoferax* as well as *Geobacter*. Here we examined whether *R*. *ferrireducens* could establish interspecies electron transfer in co-cultures with 2 DIET methanogens (*M*. *harundinacea*, *M*. *barkeri*) or with 2 strict hydrogenotrophic methanogens (*M*. *hungatei* and *M*. *formicicum*). We expected that this efficient anode-respiring bacterium^25^ would prefer DIET syntrophic partners to H_2_-utilizing partners. *R*. *ferrireducens* cannot utilize ethanol, therefore these co-cultures were provided with glucose (5 mM) as sole electron donor^33^. On glucose, *Rhodoferax* acts a respiratory organism and could not oxidize this substrate in the absence of an electron acceptor^34^. In these co-cultures, methane was used as a proxy for syntrophic metabolism. In order to estimate electron recovery from glucose, volatile fatty acid accumulation was determined during stationary phase. All co-cultures consumed the 5 mM glucose added (<4 µM detected after 270 days). Product recoveries varied significantly in *Rhodoferax* co-cultures with DIET-methanogens versus co-cultures with hydrogenotrophic methanogens (Fig. 4 and 4-inset). By comparing methane production in co-cultures with *Rhodoferax*, it was evident that *M*. *harundinacea* was the most effective at accumulating methane followed by *Ms*. *barkeri* and then strict hydrogenotrophs (Fig. 4). *Rhodoferax* in co-culture with *Methanothrix* had the highest total electron recovery (45%) with all electrons being recovered as methane, and none as acetate (Fig. 4-inset). The electrons not accounted for in products, are likely assimilated into biomass, typical of methanogenic metabolisms^35^. The *Rhodoferax* co-cultured with *Methanosarcina* was 3-fold less effective at recovering electrons into products (14%; Fig. 4-inset), yet the majority of the electrons were recovered as methane (12%) and only traces as acetate (2%). On the other hand, both co-cultures of *Rhodoferax* with strict hydrogenotrophs resulted in low electron recoveries as methane (ca. 5%) and more as acetate (18%) indicating hydrogenotrophic methanogens do not thrive in partnership with *Rhodoferax*. These results indicate that *Rhodoferax* favors interactions with DIET-methanogens rather than strict hydrogenotrophic methanogens. However, we do not know how *Rhodoferax* releases electrons to DIET methanogenic partners, although hints about its EET metabolism have been projected from genome screening^34,36^. The genome of *R*. *ferrireducens* contains 45 putative c-type cytochromes^34^ and the entire Mtr-pathway suggesting *R*. *ferrireducens* may be doing EET similar to *Shewanella*^36,37^. It remains to be tested whether this pathway is also used for DIET syntrophy with methanogens.

### Multiheme *c*-type cytochromes were not required for DIET or cathodic EET

**Figure 5:**
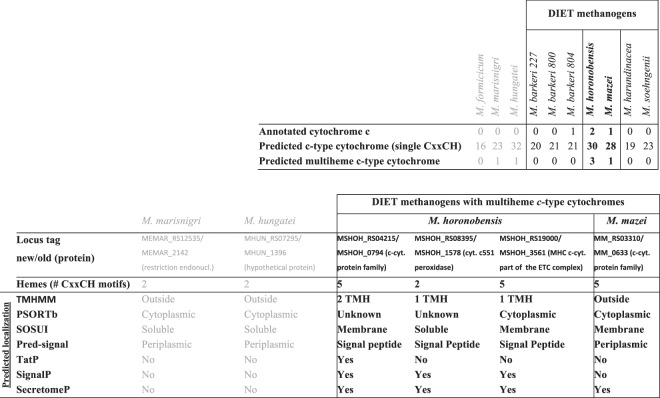
Predicted *c*-type cytochromes in methanogens tested for DIET. c-type cytochrome heme biding sites in 10 species of methanogens as predicted by CxxCH motif and/or annotated, this includes multiheme cytochromes; the predicted localization of the multiheme c-type cytochromes according to various bioinformatics tools. PSORTb - subcellular localization prediction tool^58^; TMHMM - prediction of transmembrane helices in proteins^59^; SOSUI- classification and secondary structure prediction system for membrane proteins^60^; SignalP - location of signal peptide cleavage sites, which is Archaea specific^61^; Pred-Signal - prediction of signal peptides in Archaea^62^; TatP - prediction of Twin-arginine signal peptide cleavage sites^63^; SecretomeP - prediction of non-classical protein secretion^64^.

Many studies have shown that multi-heme c-type cytochromes (MHC) are important for extracellular electron transfer^38^ including EET during DIET^1,3,12,19,39^. However, MHCs are neither ubiquitous nor restricted to DIET-methanogens (Fig. 5). Of the hydrogenotrophic methanogens tested, *M*. *hungatei* and *M*. *marisnigri* contained potential multiheme cytochrome proteins (Fig. 5), apparently localized in the cytoplasm (Fig. 5). Of the DIET-methanogens, 5 out of 7 species did not contain MHCs including all of the *Methanosarcina barkeri* strains and both *Methanosaetaceae* species (*M*. *harundinacea* and *M*. *soehngenii*). *M*. *horonobensis* and *M*. *mazei* were the only 2 species with MHC apparently localized on the membrane or secreted (Fig. 5). *M*. *mazei’s* predicted MHC was detected within the surface/membrane-bound fraction by biochemical testing and predicted to be secreted extracellularly through leaderless secretion^40^ where it is suggested to join a membrane-bound complex containing flavoproteins and iron-sulfur flavoproteins^41^.

Our hypothesis was that if *M*. *mazei* required its MHC for extracellular electron transfer, cells without it would be unable to interact with a DIET syntroph or with a poised electrode. This approach was previously used to determine *Geobacter’s* necessity for cell surface MHCs during EET to electrodes^42^, iron-oxide minerals^22^ and DIET-partners^1,3,12^.

**Figure 6:**
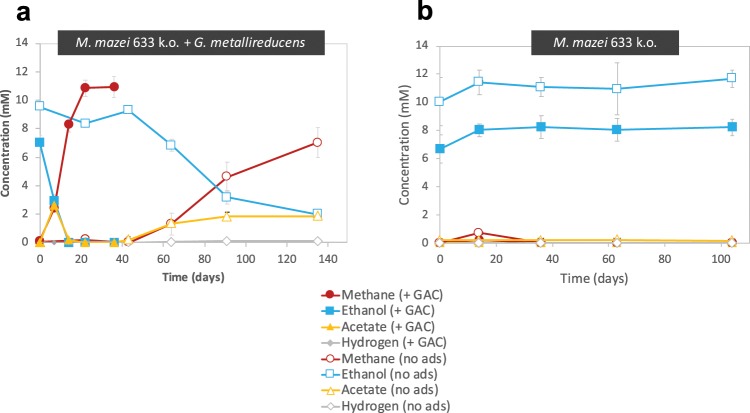
A genetically manipulated *Methanosarcina mazei* (strain 633k.o.), lacking the gene encoding a multiheme c-type cytochrome (MM_633), was tested for ethanol metabolism as single species (**a**) and in co-culture (**b**) with *G*. *metallireducens.* Incubations were carried out with or without the addition of electrically conductive GAC. The whiskers represent standard deviations of triplicate incubations (n = 3) and if invisible, they are smaller than the symbol.

To test this hypothesis, we used a knock-out mutant of *M*. *mazei* (633k.o.) in which the gene (MM_0633) encoding for the putative multiheme *c*-type cytochrome was deleted^24^. The deletion mutant showed no phenotypic variability to the wild type when growing on its typical substrates (methanol and acetate)^24^. To determine whether this MHC was required to receive DIET electrons from *Geobacter*, *M*. *mazei* 633k.o. was incubated with *G*. *metallireducens* in syntrophic media with ethanol (Fig. 6). Methane production and ethanol oxidation progressed similar to wild type control incubations (Fig. 6) demonstrating that this MHC is not required for DIET.

Recently, it was reported that *M*. *mazei* was not electroactive and incapable to retrieve electrons from a poised cathode at −700 mV (vs. SHE)^43^. However, these experiments were carried out under conditional typically associated with electrochemical H_2_-generation^44^, and for a time frame of only 3 days, which is too short to test for the formation of electrical contacts for direct electromethanogenesis^11,45^. In fact, the authors observed only the growth of strict hydrogenotrophic methanogens on cathodes poised at −700 mV^43^. Strict hydrogenotrophic methanogens have low H_2_-thresholds (ca. 6 nM)^46^, unlike *M*. *mazei*, which grows poorly on H_2_, and has a high H_2_-threshold (ca. 300 nM) like all other *Methanosarcinales*^46^. Unlike the study by Meyer *et al*.^43^, our experiements were run at −400 mV vs. SHE, condition that does not allow for abiotic electrochemical H_2_ accumulation even after several months^11^. In a recent study, we showed that a cathode at −400 mV could not be used as sole electron donor by H_2_-utilizing methanogens like *Methanobacterium formicicum*, but it was used by a strain capable of direct electron uptake (via DIET) - *Methanosarcina barkeri* strain MS^11^.

**Figure 7:**
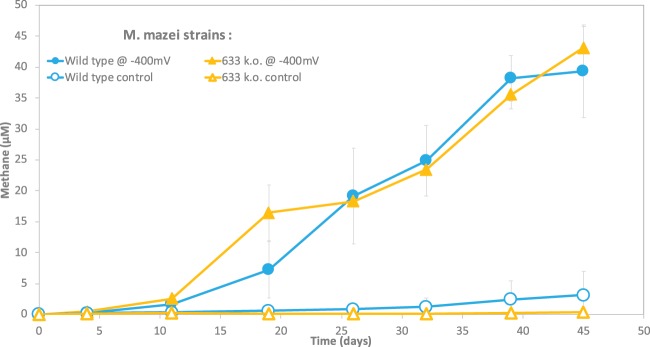
*M*. *mazei* strains (wild type and MHC k.o.) incubated with cathodes as sole electron donor. The cathode was either poised at −400 mV versus SHE (closed symbols), or not poised (empty symbols).

Here we tested whether another *Methanosarcina*, *M*. *mazei* could carry out direct electron uptake from a cathode at −400 mV. We compared a wild type *M*. *mazei* and an MHC deletion mutant of *M*. *mazei* (633k.o.) to see whether the absence of its one and only MHC impacts EET from a cathode. Both *M*. *mazei* strains with and without the multiheme cytochrome were incubated with a cathode poised at a voltage of −400 mV (vs. SHE), unfavorable for the H_2_-evolution reaction^11^. Control experiments were run alongside, without applying a voltage at the cathode, to verify whether methanogenesis can be induced by carry-over substrates. Only in experiments with a poised cathode, methane production proceeded effectively for 633k.o. and wild type *M*. *mazei* (Fig. 7), showing that the MHC is not required for electron uptake from a cathode. Previously, we observed that *M*. *horonobensis* which contains the highest number of MHCs among DIET-methanogens was also unable to use a cathode as electron donor^11^. Combined, these results disprove the hypothesis that *Methanosarcina* species require a multiheme *c*-type cytochrome for extracellular electron uptake.

**Figure 8:**
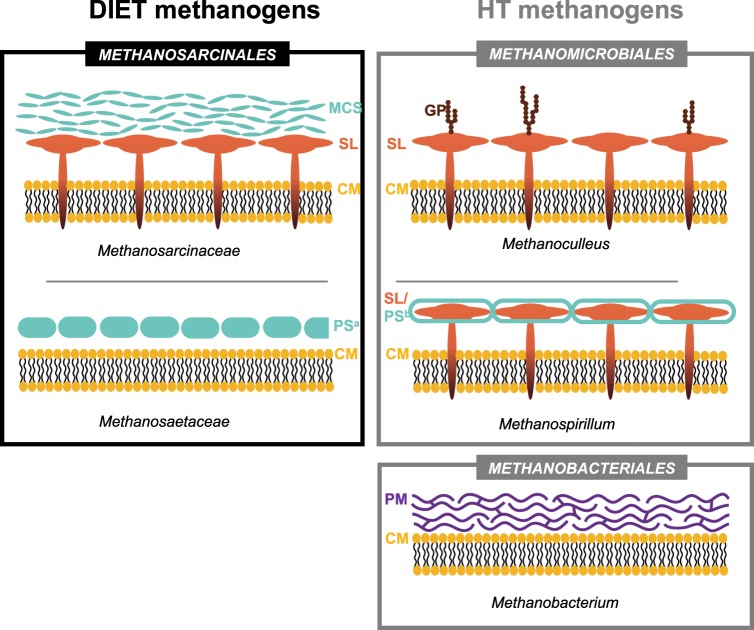
Representative cell envelope structures of DIET-methanogens (order *Methanosarcinales*) in contrast with hydrogen transfer (HT)-methanogens (*Methanobacteriales*; *Methanomicrobiales*). Abbreviations: DIET (direct interspecies electron transfer); HT (interspecies hydrogen transfer); MC (methanochondroitin); SL (S-layer); CM (cell membrane); protein sheath (PS); protein sheath amyloid type (PS^a^); protein sheet S-layer type (PS^b^); GP (glycosylated protein); PM (pseudomurein).

For *Methanosarcinales* involved in EET/DIET, the first barrier for electrons to enter a cell is the cell envelope. Thus, for DIET to take place, the cell surface of *Methanosarcinales* is anticipated to harbor charge transferring molecules. Methanogens exhibit very different cell envelopes and among the methanogens examined in this paper, as many as five types of distinct cellular surface composition have been observed (Fig. 8).

DIET-associations have now been demonstrated strictly with methanogens of the order *Methanosarcinales* including members of the families *Methanosarcinaceae* (*Methanosarcina*-genus) and *Methanosaetaceae* (*Methanothrix* and *Methanosaeta*). What distinguishes the *Methanosarcina* genus from all other methanogens is a thick methanochondroitin sulfate (MS) layer (Fig. 8), which is steadily represented on cell surfaces grown at low osmolarity^47^. Until now, DIET has been only demonstrated under freshwater conditions when *Methanosarcina* cells would be coated by methanochodroitin sulfate (MCS). MCS is an exopolysacharide resembling chondroitin sulfate in eukaryotes^48^ where it confers conduction via axonal length^49^. It is typical of exopolysaccharides like MCS to absorb metals^50^ or even trap redox cofactors and* c*-type cytochromes^51^, thus the embedded redox centers within the surface matrix may confer very different electric properties. What distinguishes the cell surface of *Methanosaeta/Methanothrix* genus from other methanogens is that only a protein sheet is delineating the cell surface from the environment^52^ (Fig. 8). The protein sheet was recently described in *Methanosaeta thermophila* to be composed of amyloid proteins^53^. Amyloid proteins are known to cluster together, while binding peptides^54^, and concentrating metal ions^55^. For example, the protein sheet of *Methanothrix shoeghenii* was described to concentrate metal ions like iron, copper, nickel and zinc^56^.

The surface structures of DIET methanogens of the order *Methanosarcinales*, although different in structure, have a shared attribute in binding/traping metal-ions to the cell surface, which we hypothesize to play a role in extracellular electron uptake by these Archaea.

Of the strict H_2_-utilizing methanogens, we observed that members of *Methanomicrobiales* and one *Methanobacteriales* were incapable to interact by DIET with electrogens. Typically, the membrane of these two groups are delineated from the environment by one single layer made either of pseudomurein (*Methanobacteriales*) or glycosylated S-layer proteins (*Methanomicrobiales*)^57^. *Methanospirillum* which is coated by an S-layer protein sheath was often compared to the protein sheet of *Methanothrix* (Fig. 8). However, the S-layer protein sheath of *Methanospirillum* traps less metal ions (2–5 fold less) than that of *Methanothrix*^56^.

These differences in surface biology may provide DIET-methanogens with a specific advantage to retrieve electrons from the extracellular environment and consequently an ecological niche where they outcompete H_2_-utilizers (e.g. mineral rich environments).

## Conclusion

The incidence of *Geobacter* in methanogenic environments is often used as signature for direct interspecies electron transfer. However, only two (*G*. *metallireducens*, *G*. *hydrogenophilus*), which were the most electroactive species out of seven *Geobacter* species tested were previously shown to pair succesfully with *Methanosarcinales*. On the other hand these two *Geobacter* were previously shown to be unable to pair with methanogens of the orders *Methanobacteriales* (*Methanobacterium formicicum*) and a *Methanomicrobiales* (*Methanospirillum hungatei*). Here, we have expanded the list of DIET partnerships between *Geobacter* and *Methanosarcinales* by six additional combinations; and confirm using another *Methanomicrobiales* (*Methanoculleus marisnigri*) that these electrogenic *Geobacter* cannot pair with a strict hydrogenotrophic methanogens. Additionally, we show that DIET may favor another effective electrogen outside the *Geobacter*-cluster - *Rhodoferrax ferrireducens*. We observed that similar to the two electrogenic *Geobacter*, *Rhodoferax ferrireducens* promoted methanogenesis with *Methanothrix* and *Methanosarcina* and not with two strict hydrogenotrophic methanogens of the orders *Methanobacteriales* and *Methanomicrobiales*.

All *Methanosarcinales* tested formed metabolically active DIET consortia with *G*. *metallireducens*. Similar to other microorganisms involved in extracellular electron transfer, *Methanosarcinales* were anticipated to require MHCs. Here we documented that MHCs are infrequent within DIET-capable *Methanosarcinales* and unrestricted to DIET-methanogens. Moreover, a deletion of the MHC in one of the two MHC-containing *Methanosarcinales* (*M*. *mazei*) did not impact its ability to retrieve extracellular electrons from a DIET-partner or from an electrode. These data confirmed that extracellular electron uptake in *Methanosarcina* did not require MHCs, prompting  a quest for cell surface constituents required for EET.
